# Vitamin D Regulates the Microbiota to Control the Numbers of RORγt/FoxP3+ Regulatory T Cells in the Colon

**DOI:** 10.3389/fimmu.2019.01772

**Published:** 2019-07-30

**Authors:** Margherita T. Cantorna, Yang-Ding Lin, Juhi Arora, Stephanie Bora, Yuan Tian, Robert G. Nichols, Andrew D. Patterson

**Affiliations:** ^1^Department of Veterinary and Biomedical Science, The Pennsylvania State University, University Park, PA, United States; ^2^Center for Molecular Immunology and Infectious Disease, The Pennsylvania State University, University Park, PA, United States; ^3^CAS Key Laboratory of Magnetic Resonance in Biological Systems, State Key Laboratory of Magnetic Resonance and Atomic and Molecular Physics, National Centre for Magnetic Resonance in Wuhan, Wuhan Institute of Physics and Mathematics, University of Chinese Academy of Sciences, Wuhan, China

**Keywords:** vitamin D, T regulatory (T reg) cells, inflammatory bowel diseases, microbiota, gnotobiotic mice

## Abstract

The active form of vitamin D (1,25(OH)_2_D) suppresses experimental models of inflammatory bowel disease in part by regulating the microbiota. In this study, the role of vitamin D in the regulation of microbe induced RORγt/FoxP3+ T regulatory (reg) cells in the colon was determined. Vitamin D sufficient (D+) mice had significantly higher frequencies of FoxP3+ and RORγt/FoxP3+ T reg cells in the colon compared to vitamin D deficient (D–) mice. The higher frequency of RORγt/FoxP3+ T reg cells in D+ colon correlated with higher numbers of bacteria from the *Clostridium* XIVa and *Bacteroides* in D+ compared to D– cecum. D– mice with fewer RORγt/FoxP3+ T reg cells were significantly more susceptible to colitis than D+ mice. Transfer of the cecal bacteria from D+ or D– mice to germfree recipients phenocopied the higher numbers of RORγt/FoxP3+ cells and reduced susceptibility to colitis in D+ vs. D– recipient mice. 1,25(OH)_2_D treatment of the D– mice beginning at 3 weeks of age did not completely recover RORγt/FoxP3+ T reg cells or the *Bacteriodes, Bacteriodes thetaiotaomicron*, and *Clostridi*um XIVa numbers to D+ values. Early vitamin D status shapes the microbiota to optimize the population of colonic RORγt/FoxP3+ T reg cells important for resistance to colitis.

## Introduction

The intestinal microbiota is critical for maintenance of gut homeostasis and experimental colitis fails to develop in germfree (GF) mice. The composition of the microbiota regulates diseases of the gastrointestinal (GI) tract; including, celiac and inflammatory bowel diseases (IBD) (1–3). IBD is associated with decreased diversity and dysbiosis of the microbiota and the decreased abundance of *Firmicutes* and *Bacteroides* and increased *Gammaproteobacteria* (4). Th17/Th1 cells that target the normal microbiota contribute to colitis (5). Conversely, FoxP3-expressing CD4+ regulatory T (T reg) cells play a crucial role in suppressing IBD (6, 7). The microbiota determines the types of T cells that are found in the GI tract (8, 9). The presence of segmented filamentous bacteria in a mouse colony is sufficient for the development of robust Th17 cells (8, 9). In addition, *Bacteroides* and *Clostridium* species have been found to suppress experimental colitis via the induction of T reg cells (10, 11). Colitis is a result of a T cell response to the microbiota and dysbiosis of the microbiota.

Vitamin D is an important regulator of GI homeostasis. Vitamin D and or vitamin D receptor (VDR) deficiency increases the susceptibility of mice to several different models of experimental colitis (5, 12–14). The active form of vitamin D (1,25(OH)_2_D, 1,25D) suppresses experimental colitis (5, 12–14). The mechanisms by which vitamin D regulates experimental colitis includes a role for vitamin D in shaping the microbiota (15). Th17 cells are direct targets of 1,25D (16, 17). *In vitro*, 1,25D inhibited Th17 cells and induced FoxP3+ T reg cells (17, 18). 1,25D treatment of mice induced the expansion of FoxP3+ T reg cells *in vivo* (19). However, expression of the VDR is not required for normal development of peripheral FoxP3+ T reg cells (14). Vitamin D inhibits Th17 cells and induces peripheral T reg cells to maintain GI homeostasis.

Within the colon are a unique population of T reg cells that also express the transcription factor retinoic acid receptor–related orphan receptor (ROR)γt and are RORγt/FoxP3+ T reg cells (20). The colon also contains FoxP3+ single positive T reg cells and GATA3/FoxP3+ T reg cells which are derived from the thymus (21). The majority of the T reg cells found in the colon are RORγt/FoxP3+ T reg cells and this population of cells are low/absent in GF mice (22, 23). Microbial transplants into GF mice can recover the RORγt/FoxP3+ T reg cells (22, 23). Mice without RORγt/FoxP3+ T reg cells developed severe inducible experimental colitis (22, 23). RORγt/FoxP3+ T reg cells from the colon were better suppressors of colitis than splenic CD25+ T reg cells in the T cell transfer colitis model (23). The RORγt/FoxP3+ T reg cells in the colon are important for the maintenance of GI homeostasis and to prevent the development of colitis.

The effect of vitamin D on microbiota-dependent RORγt/FoxP3+ T reg cells were determined in the colons of vitamin D sufficient (D+) and vitamin D deficient (D–) mice. D– mice had significantly fewer total FoxP3+ T reg cells in the colon. The reduction in FoxP3+ T reg cells was due to reduced numbers of RORγt/FoxP3+ T reg cells, which were 80% of the FoxP3+ T reg cells in the colon. Microbial transplants from D+ and D– mice into GF mice resulted in lower RORγt/FoxP3+ T reg cells in the D– vs. the D+ recipients. D– microbiota had fewer numbers of *Bacteroides* and *Clostridium* XIVa species that were correlated significantly with the RORγt/FoxP3+ T reg frequencies. 1,25D treatments of D– mice were ineffective for inducing RORγt/FoxP3+ T reg cells or increasing *Bacteroides* or *Clostridium* XIVa numbers to the D+ values. D– mice and GF recipients of D– microbiota were more susceptible to dextran sodium sulfate (DSS) induced colitis than their D+ counterparts. Vitamin D status is an important factor that shapes the microbiota and impacts the expansion and development of protective colonic RORγt/FoxP3+ T reg cells.

## Methods

### Mice

C57BL/6 mice were originally from Jackson Labs (Bar Harbor, MN) and maintained at the Pennsylvania State University (University Park, PA). All mice used were housed within the same rooms in the animal facility. Mice were fed purified diets made in the lab as described that either contained vitamin D (D+) or did not (D–) (24). GF C57BL/6 mice were bred and maintained at the Pennsylvania State University gnotobiotic animal research facility. For some experiments, 1,25D was started in the diets of 3–5 week old mice (25 ng/d until 5 weeks of age) and mice that were >5 weeks old were fed 50 ng/d 1,25D exactly as described (24). For microbial transplantation experiments (two independent experiments), the 4 week old GF mice were gavaged with 100 μl/10 μg cecal contents from D+ or D– mice, and used for experiments after 2 weeks (25). All of the experimental procedures were approved by the Institutional Animal Care and Use Committee at the Pennsylvania State University.

### Cell Isolation and Flow Cytometry

Colon lamina propria (LP) cells were isolated as described previously and stained for flow cytometry (26). Briefly, the colon tissues were washed and cut into 1–1.5 cm sections incubated in Hanks' Balanced Salt Solution (Sigma-Aldrich, St. Louis, MO) with 5 mM EDTA at 37°C, and then digested in RPMI-1640 containing 1 mg/ml collagenase type 1 (Worthington, Lakewood, NJ) and 10% FBS at 37°C for 1 h in a shaking incubator. The cells were collected from the interface of 40/80% Percoll gradients (Sigma-Aldrich). The fluorescent dye conjugated-antibodies listed below were used for flow cytometry: CD3 (clone 145-2C11), CD4 (clone GK1.5) from Biolegend (San Diego, CA), CD45.2 (clone 104) from BD Biosciences (San Jose, CA), FoxP3 (clone FJK-16s), RORγt (clone B2D) from eBioscience (San Diego, CA). The cells were fixed and permeabilized using kits for intracellular staining (eBioscience) and the manufacturer's instructions. All data were collected on a BD Fortessa LSRII (BD Biosciences) and analyzed with FlowJo software (TreeStar, Ashland, OR).

### Gut Microbiota Analysis

The bacterial DNA in the cecal contents was extracted using the E.Z.N.A stool DNA isolation kit (OMEGA Bio-tek, Norcross, GA) according to the manufacturer's instructions. A 100 μl aliquot of the isolated bacterial DNA was created with a bacterial DNA concentration of 10 ng/μl. PCR was completed as previously described (27). Bacteria were then analyzed by qPCR using primers targeted at 16S ribosomal RNA gene (28, 29). Primers for all of the species in the *Clostridium* clusters XIVa, IV, and XVIII were used and listed in Supplementary Table 1 (28, 29). qPCR assays were carried out using SYBR Green qPCR Master Mix on a StepOnePlus real-time PCR system (Thermo Fisher Scientific, Waltham, MA) (30). The results were normalized to 16S ribosomal (universal) DNA sequences and expressed as the relative difference compared to D+ control group using the ΔΔC_T_ method.

16S rRNA sequencing was performed using the Illumina MiSeq platform by the Pennsylvania State University Genomics Core Facility. Data analysis was done using mothur and aligned with the SILVA databases as described previously (27, 30–32). The raw data can be accessed on the National Center for Biotechnology Information (NCBI) with the accession number PRJNA506977.

### DSS Colitis

Mice were administered DSS (MP Biomedicals, Solon OH) for 5 days, after which mice were given regular drinking water. Animals were weighed daily, and monitored for rectal bleeding, diarrhea, and signs of morbidity. Because of differences in initial starting weights (**Figure 7**) the females received 3.5% DSS and the males received 4% DSS. Based on their identical initial starting weights the male and female GF recipients of the D+ or D– microbial transplants received 3.5% DSS (Figure 8). Diarrhea was scored based on a scoring system as described (33). Briefly: 0- firm normal stool, 2- semi-formed stool, and 4- liquid stool that adhere to the anal region (33). The entire colon from cecum to anus was removed and the length was measured and reported as colon length. Distal colon was fixed in 10% formalin, sectioned, and stained with hematoxylin and eosin (Pennsylvania State University Animal Diagnostic Laboratory). Histological analysis for severity of inflammation, injury, and crypt damage was performed blinded by two independent investigators as described previously (13, 15, 34). Inflammation was scored from 0–3 (0 = none, 1 = slight, 2 = moderate, 3 = severe). Injury was scored from 0–3 (0 = none, 1 = mucosal, 2 = mucosal and submucosal, 3 = transmural). Crypt damage was scored for 0–4 (0 = none, 1 = basal 1/3 damaged, 2 = basal 2/3 damaged, 3 = only surface epithelium intact, 4 = entire crypt and epithelium lost). Each score was added to obtain a total histology score where the maximum score was 10.

### Statistics

Statistical analyses were performed with PRISM software by (GraphPad, La Jolla, CA). Two-tailed Student's *t*-test, Mann-Whitney tests or one-way ANOVA with Kruskal–Wallis or Bonferonni *post hoc* tests were used for data analyses. ^*^P < 0.05 ^**^P < 0.01, ^***^P < 0.001, ^****^P < 0.0001 were used to indicate significance in the figures.

## Results

### Reduced FoxP3+ and RORγt/FoxP3+ Colonic Regulatory T Cells in D– Mice

**Figure 1:**
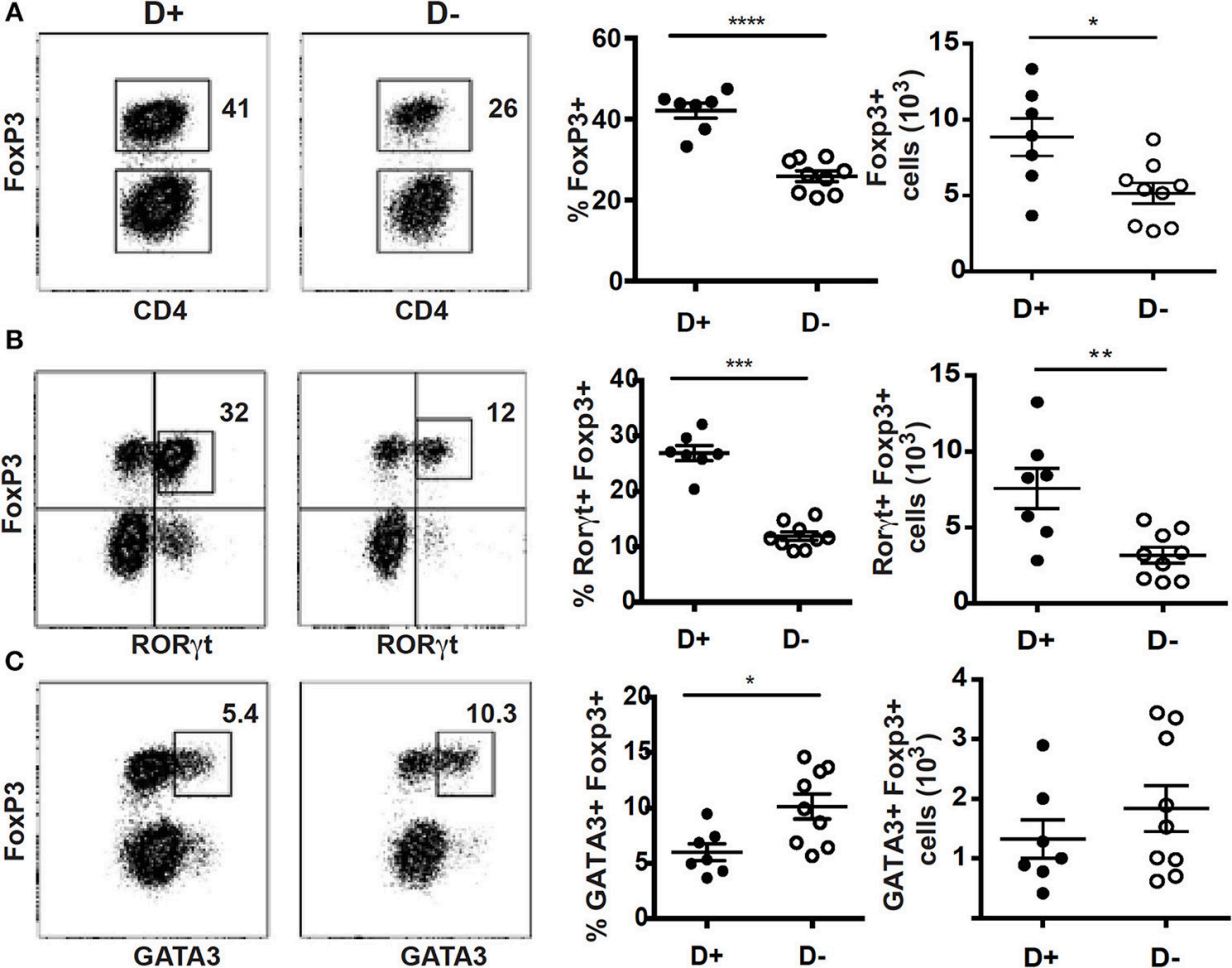
Reduced frequencies of FoxP3+ and RORγt/FoxP3+ T reg cell populations in the colon of D– mice. Dot plot, gating, frequencies and numbers of the CD4+ cells that are **(A)** FoxP3+, or **(B)** RORγt+/FoxP3+, and **(C)** GATA3+/FoxP3+ positive in the D+ and D– colons. Values are mean ± SEM of two combined experiments and *n* = 7–9 mice/groups. Two-tailed Student's *t*-tests was used for determining significance. ^*^P < 0.05, ^**^P < 0.01, ^***^P < 0.001, ^****^P < 0.0001.

Mice with defects in vitamin D metabolism or without the VDR develop dysbiosis of the microbiota, which was associated with alterations in mucosal immune function (15, 35). Here we determined the effect of changes in vitamin D on the microbially dependent colonic T reg cells. There were no significant differences in the total cells isolated from D+ and D– colon (Supplementary Figure 1A). The frequencies of αβ T cells, CD4+ and CD8+ cells were not different in D+ and D– colon LP (Supplementary Figure 1B). The D– mice had significantly fewer ILC3 cells, and reduced IL-22 compared to D+ mice [Supplementary Figure 1B; (36)]. In addition, the Th17 cells from the D– colon were unable to expand after gastrointestinal infection (36). The frequency and numbers of T reg cells in the D+ colon were significantly higher than in D– mice (Figure 1A). Most of the T reg cells in the D+ colon were RORγt/FoxP3+ (80% of the FoxP3+ cells, Figure 1B), the remainder of the T reg cells either co-expressed GATA3 or singly express FoxP3 (10% each, Figure 1B). D+ mice had significantly more RORγt/FoxP3+ T reg cells than D– mice (Figure 1B). The frequency of GATA3/FoxP3+ T reg cells was lower in D+ than D– colon (Figure 1C). However, there were no differences in the total cell numbers of GATA3/FoxP3+ T cells in the D+ and D– colons (Figure 1C). The colon of D– mice had fewer T reg cells and in particular fewer of the RORγt/FoxP3+ T reg cells in the colon than D+ mice.

### 1,25D Treatment Fails to Completely Recover Colonic RORγt/FoxP3+ T regs in D– Mice

**Figure 2:**
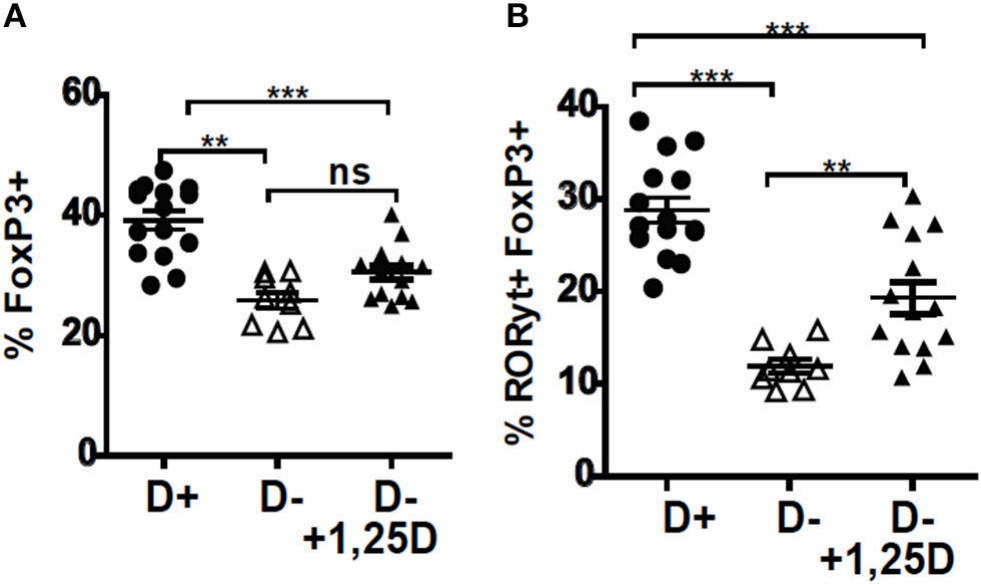
1,25D treatments were ineffective for increasing RORγt/FoxP3+ T reg cells in D– mice. 1,25D treatments were started when the mice were 3 weeks of age and continued until sacrifice at 8 weeks of age. Frequencies of **(A)** FoxP3+ and **(B)** RORγt/FoxP3+ T reg cells in the colon of D+, D–, and D– +1,25D mice. Values are the mean ± SEM of three combined experiments and *n* = 9–15 mice/group. Significance was determined using one-way ANOVA with Bonferroni's *post-hoc* test. ^**^P < 0.01, ^***^P < 0.001.

The D– mice were treated with 1,25D to determine whether 1,25D treatment could recover the T reg frequencies in the colon. Beginning the 1,25D treatments at either 5 weeks of age or 3 weeks of age significantly increased the frequencies of RORγt/FoxP3+ T reg over those in the D– mice (Supplementary Figure 2 and Figures 2A,B). However, 1,25D treatment of D– mice failed to completely recover the FoxP3+ T reg or RORγt/FoxP3+ T reg frequencies to D+ levels (Figures 2A,B). D+ mice had significantly more FoxP3+ T reg and RORγt/FoxP3+ T reg than the 1,25D treated D– mice (Figure 2). 1,25D treatment of D– mice did completely recover ILC3 frequencies to those in D+ mice (36), suggesting that the failure of 1,25D to completely recover the T reg cell population was selective.

### Reconstitution of GF Mice With D– Microbiota Resulted in Fewer Colonic RORγt/FoxP3+ T reg Cells

**Figure 3:**
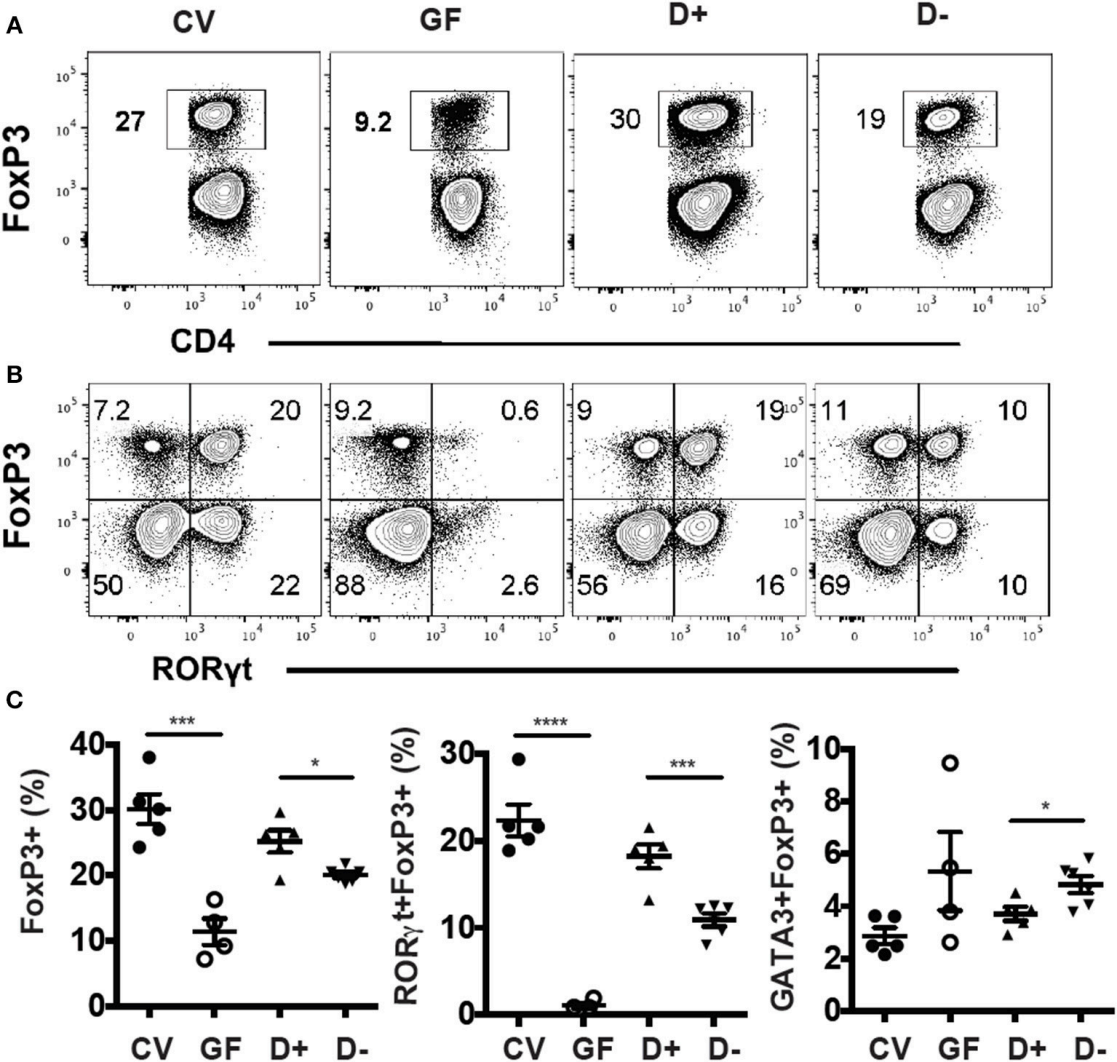
GF recipients of D+ microbiota have more RORγt/FoxP3+ T reg cells than GF recipients of D– microbiota. Flow plots and gating strategies for **(A)** FoxP3+, and **(B)** RORγt/FoxP3+ T reg cells in conventional (CV) mice, GF, D+ recipient, and D– recipient GF mice. **(D)** Frequencies of FoxP3+, RORγt/FoxP3+, and GATA3/FoxP3+ T reg cells in CV, GF, and D+ and D– recipient GF mice. Values are mean ± SEM and *n* = 4–6 mice/groups. Significance was determined using one-way ANOVA with Bonferroni *post-hoc* tests. ^*^P < 0.05, ^***^P < 0.001, ^****^P < 0.0001.

Conventional (CV) mice had significantly more colonic FoxP3+ T reg cells compared to GF mice (Figure 3A). RORγt/FoxP3+ T reg cells were almost completely absent in GF mice (Figure 3B). Colonization of GF mice with D+ cecal microbiota resulted in colonic FoxP3+ T reg cell and RORγt/FoxP3+ T reg cell frequencies that were similar to those in CV colons (Figures 3A,B). Mice that received D+ microbiota had significantly higher FoxP3+ T reg and RORγt/FoxP3+ T reg cells than mice that received D– microbiota (Figure 3C). The GATA3/FoxP3+ T reg cell frequencies were the same in GF and CV mice (Figure 3C). Recipients of D+ microbiota had fewer GATA3/FoxP3+ T reg cells than recipients of D– microbiota (Figure 3C). Microbial transplants from D+ and D– mice into GF mice phenocopied the original RORγt/FoxP3+ T reg frequencies in the D+ and D– donor mice.

### Microbial Dysbiosis in D– Mice

**Figure 4:**
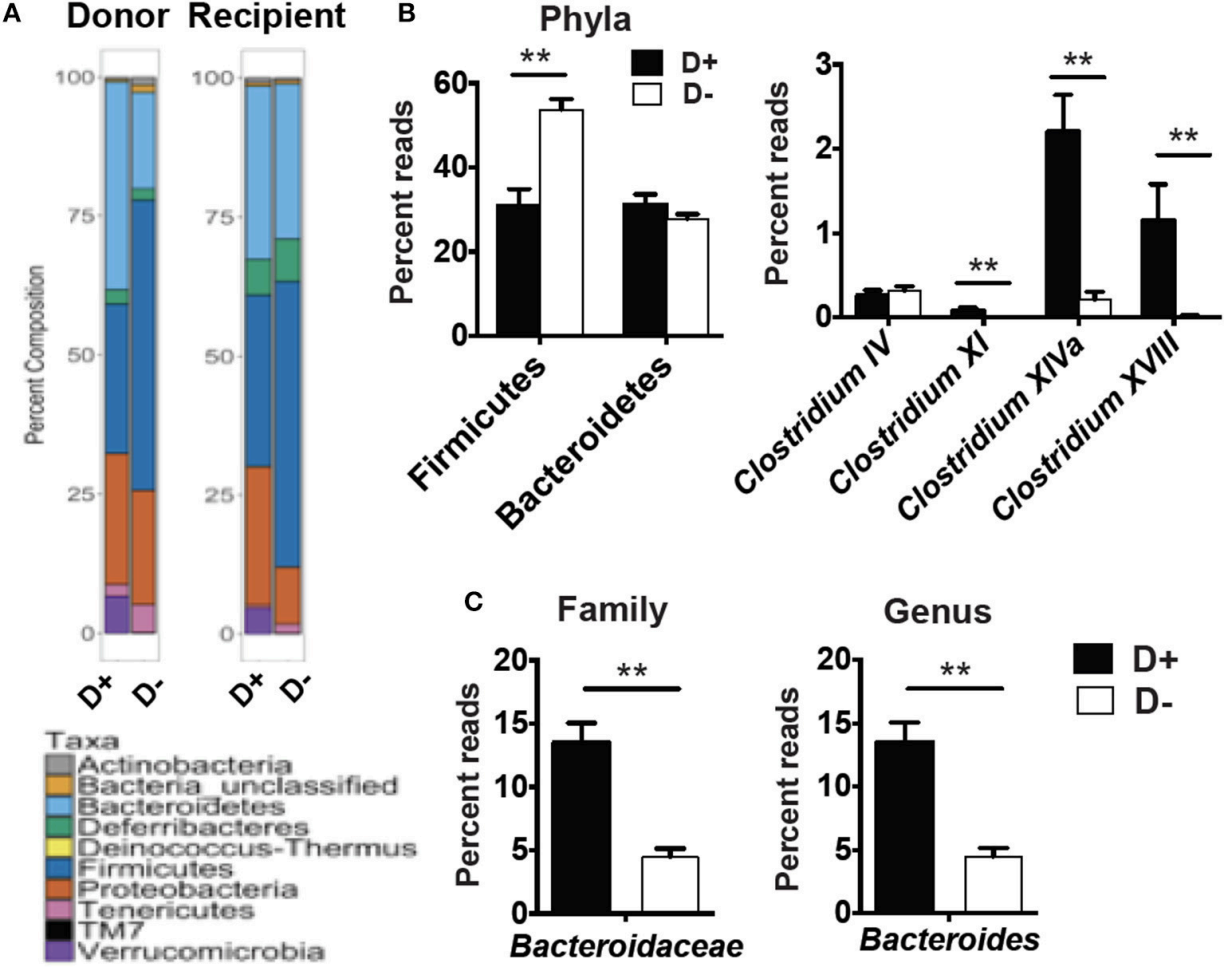
Microbial differences in the recipients of D+ and D– microbes. 16S rRNA gene sequencing analysis of GF recipients of D+ and D– cecal transplants. **(A)** Percent composition of the total reads for each phyla in the donor and recipients of one representative of two independent experiments with *n* = 2 donors/group and *n* = 5–6 recipients/group. **(B)** Reads for Phyla, and *Clostridium* cluster IV, XI, XIVa, and XVIII or **(C)** *Bacteroidaceae*, and *Bacteroides* in cecum of D+ and D– recipients. Values are the mean ± SEM of *n* = 5–6 mice per group. Significance was determined using Mann-Whitney tests. ^**^P < 0.01.

**Figure 5:**
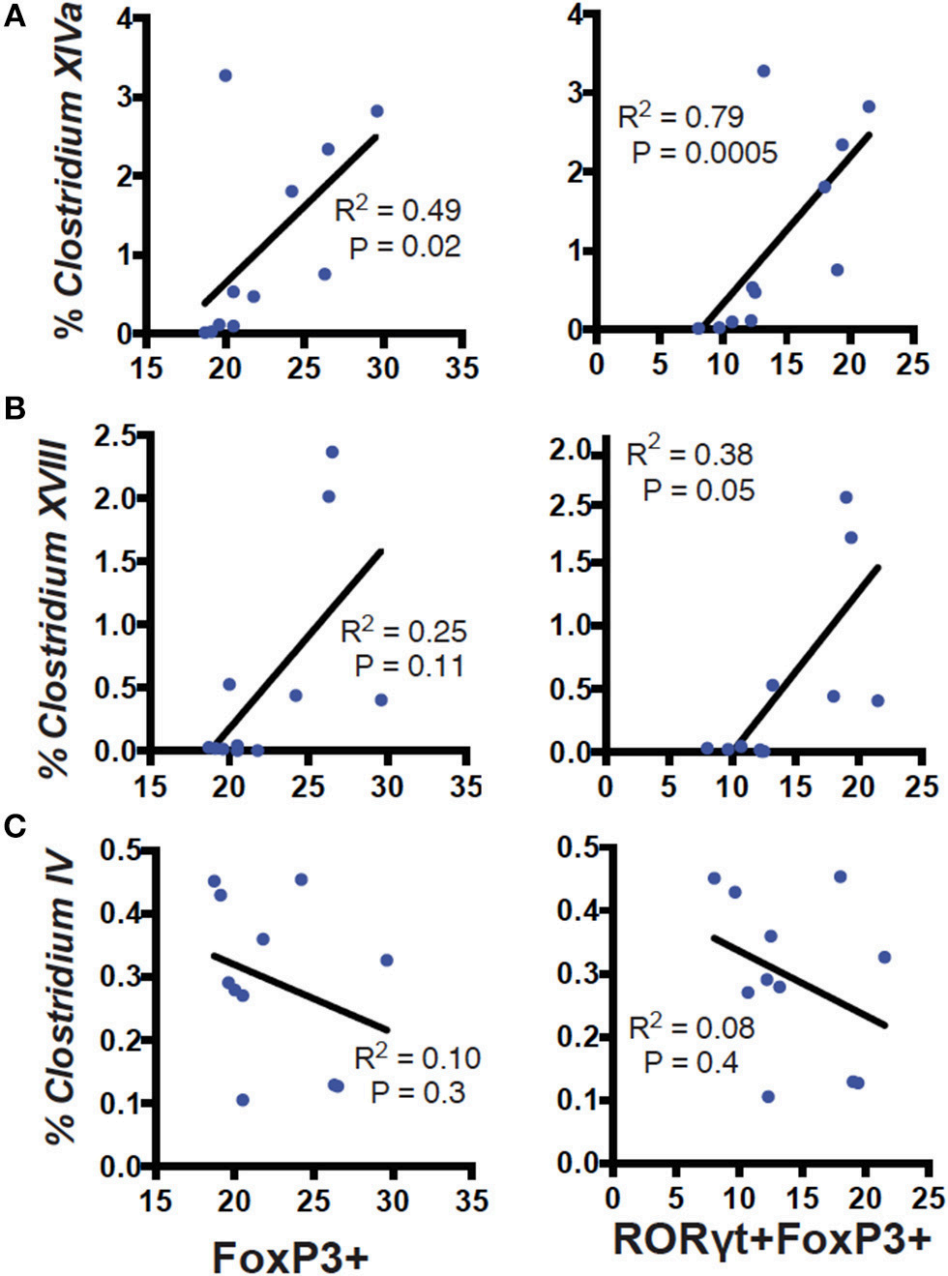
Correlation of *Clostridium* clusters with colonic T reg cells. Percent reads of *Clostridium* **(A)** XIVa, **(B)** XVIII, **(C)** IV vs. FoxP3+ (left) or RORγt/FoxP3+ (right) T reg cells in the colon of D+ and D– GF recipient mice. Values are from 11 individual mice. Spearman correlation was used to calculate the *P* values.

RORγt/FoxP3+ T reg cells develop and expand following microbial signals (23). The cecal microbial composition of the D+ and D– GF recipients were sequenced (Figure 4A). Reconstitution of GF mice with D+ microbiota resulted in the recipients having microbiota that grouped with the D+ donors as seen with generalized unifrac (Supplementary Figure 3A). Conversely, the GF mice that were reconstituted with D– microbes resulted in microbe populations that were different from the original D– donor populations (Supplementary Figure 3). The microbes in D+ and D– recipients were significantly different from one another (Supplementary Figure 3). *Firmicutes* phyla members were lower in D+ than D– recipient mice (Figure 4B). Within the *Firmicutes* phyla, D+ recipient mice had more bacteria from the *Clostridium* clusters XI, XIVa, and XVIII as compared to D– recipient mice, while the *Clostridium* cluster IV numbers were similar between groups (Figure 4B). D+ recipient mice had a larger population of the family *Bacteroidaceae* and the genus *Bacteroides* (Figure 4C). D+ recipient mice had a smaller *Firmicutes* population but a larger population of the genus *Clostridium* and *Bacteroides* than D– recipient mice. There was a significant, linear relationship between the numbers of *Clostridium* XIVa, and XVIII and the frequency of colonic RORγt/FoxP3+ T reg cells (Figures 5A,B). Conversely, the *Clostridium* cluster IV was not significantly related to RORγt/FoxP3+ T reg cells frequencies in the colon (Figure 5C). Reduced frequencies of RORγt/FoxP3+ T reg cells in D– mice were associated with reductions in *Clostridium* XIVa and XVIII numbers.

**Figure 6:**
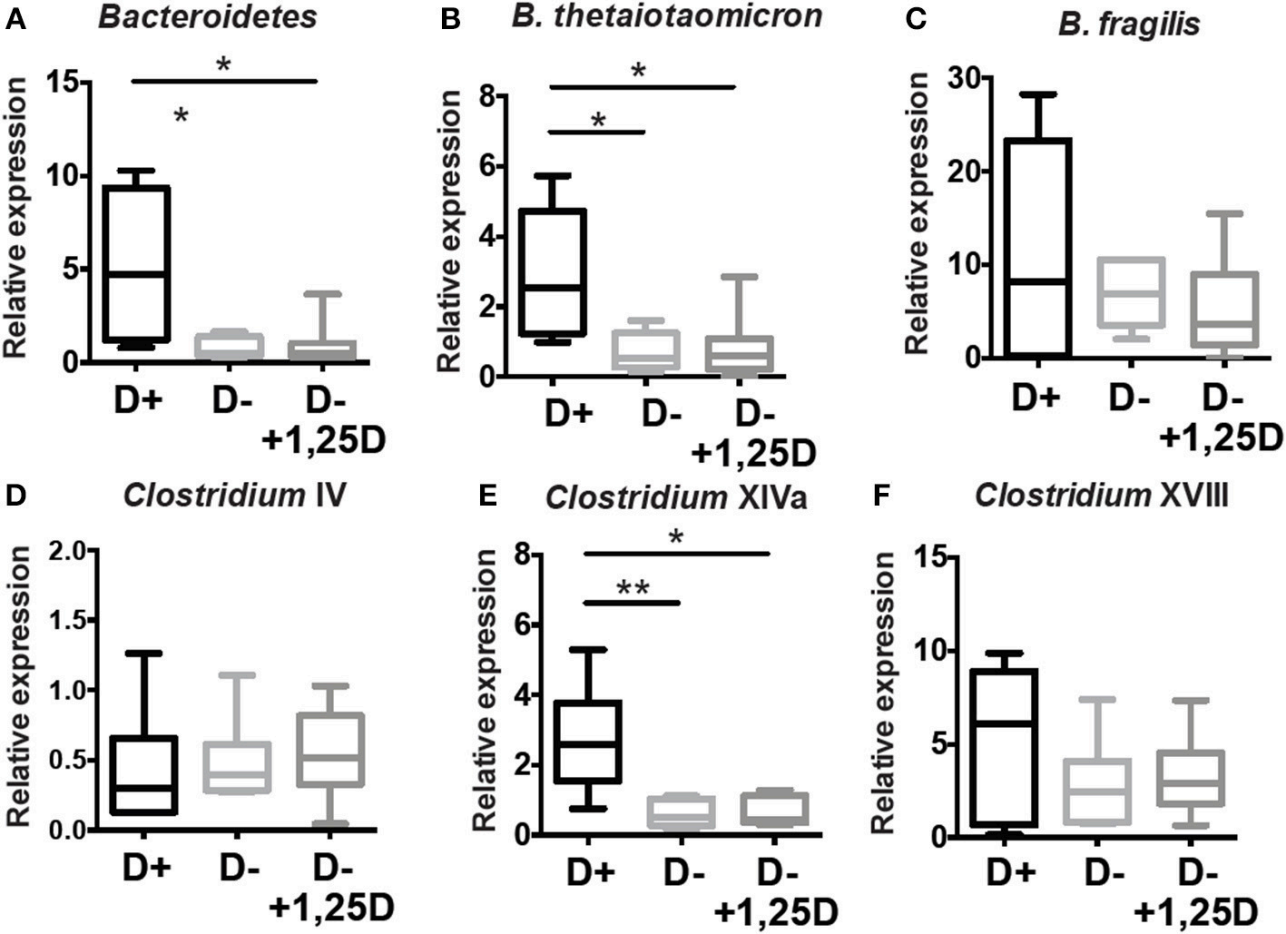
1,25D treatment of D– mice is ineffective for raising *Bacteriodetes, B.thetaiotaomicron*, and *Clostridium* XIVa to D+ levels. qPCR analysis of the proportion of **(A)** *Bacteriodetes*, **(B)** *B.thetaiotaomicron*, **(C)** *B. fragilis*, **(D)** *Clostridium* IV, **(E)** *Clostridium* XIVa, and **(F)** *Clostridium* XVII in the cecum of D+, D– and D– +1,25D mice. Values are the mean ± SEM two combined experiments and *n* = 6–14 mice/group. Significance was determined using one-way ANOVA with Kruskal-Wallis *post-hoc* tests, ^*^P < 0.05, ^**^P < 0.01.

Additional analyses of the cecal microbial composition was done in the cecum of D+, D–, and D– + 1,25D treated mice by PCR (Figure 6). D– mice had fewer *Bacteriodetes, B.thetaiotaomicron*, and *Clostridium* XIVa numbers than D+ mice (Figures 4, 6). The reduced numbers of *Clostridium* XVIII numbers in the D– microbiota found in the sequencing data was not found with PCR of expanded numbers of D+ and D– mice (Figures 4, 6). The numbers of *B. fragilis, Clostridium* IV, and *Clostridium* XVIII were similar in D+, D–, and D– +1,25D (Figure 6). The 1,25D treatments were ineffective for raising *Bacteriodetes, B.thetaiotaomicron*, and *Clostridium* XIVa numbers to D+ levels (Figure 6). Conversely, 1,25D treatments significantly decreased the *Firmicutes* population in the cecum to D+ levels (Supplementary Figure 3B). 1,25D treatment of D– mice was ineffective for restoring *Bacteriodetes, B.thetaiotaomicron*, and *Clostridium* XIVa numbers to D+ values.

The metabolites found in the cecum of mice describe the functioning of microbial communities of mice (31). OPLS-DA plots showed that the cecal metabolites were distinct between donor D+ and D– mice (Supplementary Figure 4A), recipient D+ and D– mice (Supplementary Figure 4B) and 1,25D treated vs. D+ mice (Supplementary Figure 4C). There were no consistent signatures that could explain the higher RORγt/FoxP3+ T reg cells in D+ donor and recipient mice as compared to D– donor and recipient mice or D– +1,25D mice (Supplementary Figure 4). In particular, the short-chain fatty acids (SCFA) in D+ donor cecal contents had higher propionate than the D– donors (Supplementary Figure 4A) and the D+ recipients had lower butyrate than the D– recipients (Supplementary Figure 4B). There were no differences in SCFAs in the cecal contents from D+ and D– +1,25D mice (Supplementary Figure 4C). SCFAs may not be the cause of changes in RORγt/FoxP3+ T reg populations between D+, D–, and D– +1,25D treated mice.

### Increased Susceptibility of D– Mice and GF Recipients of D– Microbiota to DSS Colitis

**Figure 7:**
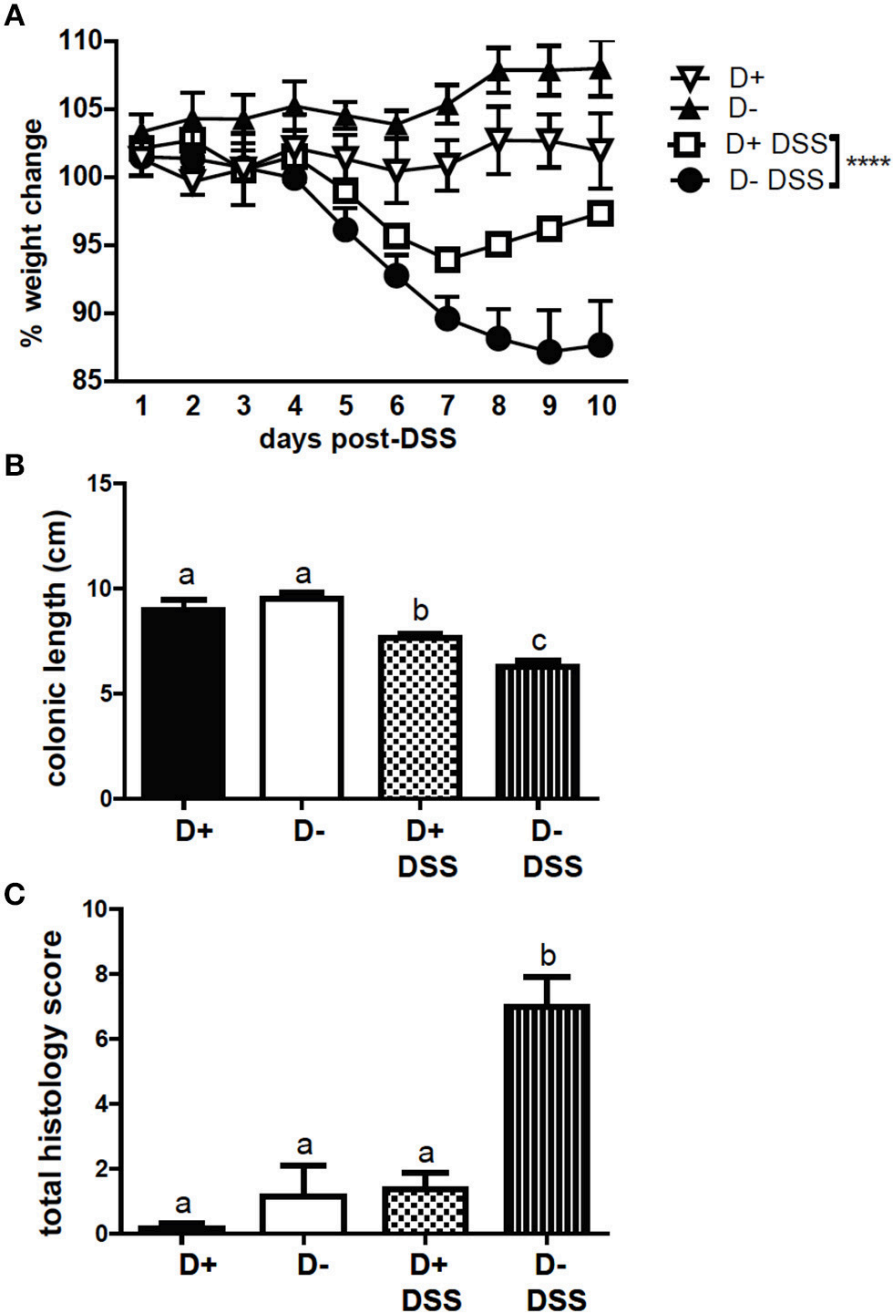
D+ mice are less susceptible to DSS colitis than D– mice. **(A)** Percent weight change, **(B)** colon length, and **(C)** total histopathology scores of untreated (*n* = 3) and DSS treated (*n* = 5–6) D+ and D– mice. Values are the mean ± SEM of *n* = 3–6 mice per group. Two-way ANOVA with Bonferroni's *post-hoc* test was used to compare **(A)** weight change between D+ DSS and D– DSS treated mice over time. ^****^P < 0.0001. One-way ANOVA with Tukey's multiple comparison was used to assess colitis symptoms **(B,C)**. Groups in **(B)** and **(C)** with different letters were significantly different from each other, *P* < 0.001.

FoxP3+ T reg cells have been shown to protect mice from DSS induced colitis (10). DSS induced weight loss in D+ and D– mice; however, D– mice lost significantly more weight than D+ mice following DSS administration (Figure 7A). The colons of D+ and D– mice shortened following 10 days of DSS (Supplementary Figure 5 and Figure 7B). D– mice had significantly shorter colons than D+ mice at day 10 post-DSS (Supplementary Figure 5 and Figure 7B). Histopathology scores from untreated D+ and D– mice were low and DSS induced inflammation, crypt damage, and injury in the colon of D– mice (Supplementary Figure 5 and Figure 7C). Inflammation, crypt damage and injury were all higher in D– mice than in D+ mice at day 10 post-DSS (Supplementary Figure 5). The total histology scores of D– mice treated with DSS was significantly higher than untreated D+ and D– mice and DSS treated D+ mice (Figure 7C). D– mice were more susceptible to DSS-induced colitis compared to D+ mice.

**Figure 8:**
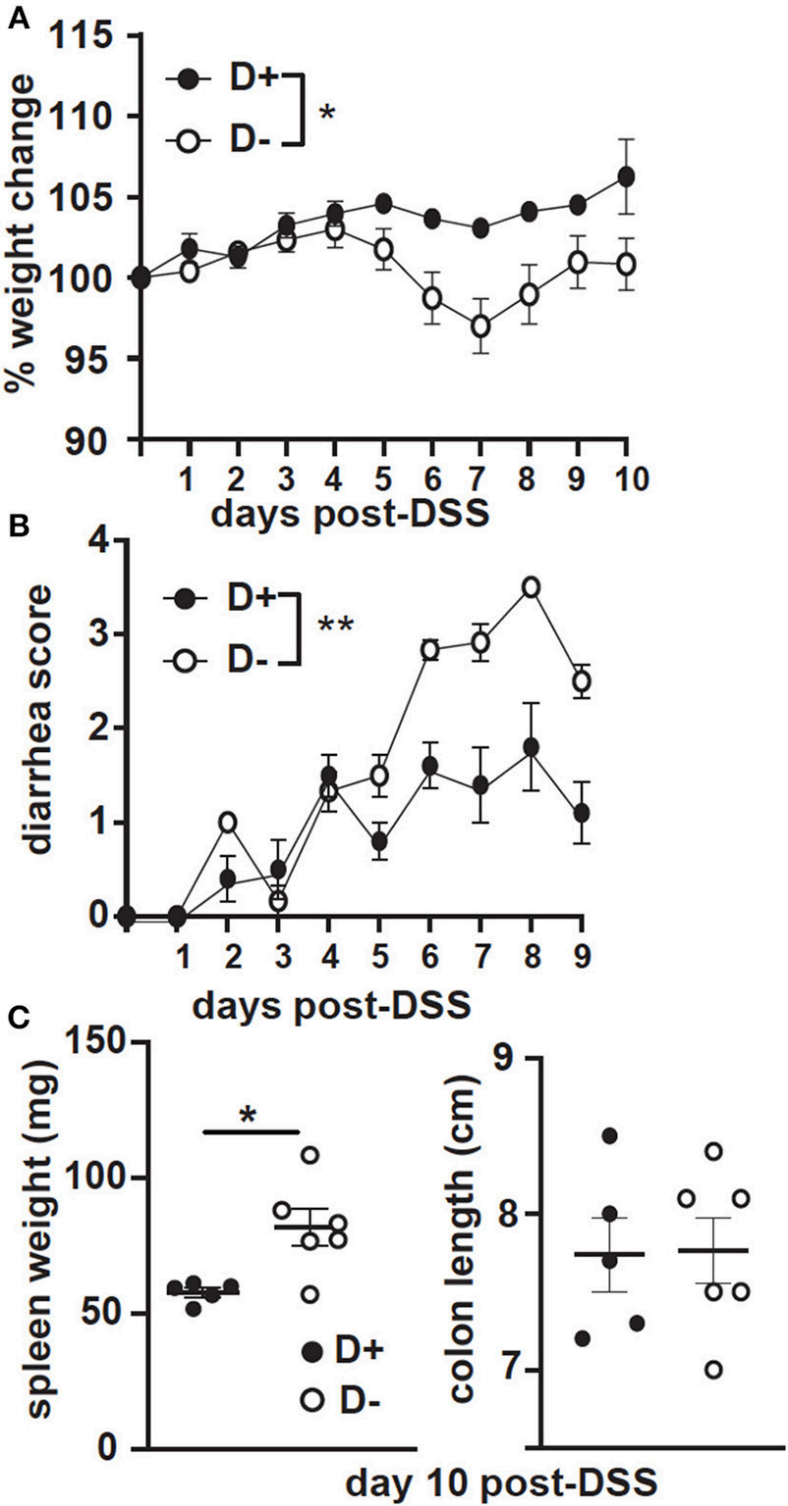
GF recipients of D+ microbiota develop milder DSS colitis as compared to GF recipients of D– microbiota. GF recipients of D+ or D– microbiota were challenged with DSS. **(A)** Percent weight change, **(B)** diarrhea scores, and **(C)** spleen weight or colon length at day 10 post-DSS. Values are the mean ± SEM of *n* = 5–6 mice per group. Significance was assessed in **(A)** and **(B)** by two-way ANOVA with Bonferroni's *post-hoc* test and by Mann-Whitney tests for **(C)**. ^*^P < 0.05, ^**^P < 0.01.

GF recipients of D+ and D– microbiota were challenged with DSS. The GF recipients of D+ microbiota largely maintained their starting weight following DSS treatment (Figure 8A). In contrast the GF recipients of D– microbiota lost significantly more weight than the GF recipients of the D+ microbiota (Figure 8A). The GF recipients of the D+ microbiota developed mild diarrhea while the GF recipients of the D– microbiota developed significantly more severe diarrhea following DSS induced colitis (Figure 8B). The colon lengths were not different, but the spleen weight was significantly heavier at day 10 post-DSS in the D– vs. D+ recipients (Figure 8C). The GF recipients of D– microbiota developed more severe DSS colitis than the GF recipients of D+ microbiota.

## Discussion

D– mice had fewer colonic RORγt/FoxP3+ T reg cells that resulted in reduced total FoxP3+ T reg cells compared to D+ mice. The cause of the reduced RORγt/FoxP3+ T reg cells was the differences in the microbiota of D+ and D– mice. Microbial transplants into GF recipients using D+ and D– cecal contents recapitulated the colonic RORγt/FoxP3+ T reg cell deficiency of the D– mice. D– mice had fewer *Clostridium* species from the XIVa cluster that have been shown to expand colonic RORγt/FoxP3+ T reg cells (10, 37). 1,25D treatments of D– mice failed to completely recover the colonic RORγt/FoxP3+ T reg cells even when started at weaning (3 weeks of age). The 1,25D treatments also failed to recover some of the microbes to D+ levels. D– + 1,25D treated mice had D– levels of *Bacteriodes, B.thetaiotaomicron*, and *Clostridium* cluster XIVa species. Colonization of GF mice with only *B.thetaiotaomicron* was enough to restore conventional numbers of RORγt/FoxP3+ T reg cells (23). Here 1,25D treatments partially restored colonic RORγt+ T reg cell frequencies but not to D+ levels. Recent data showed that D– mice took significantly longer than D+ mice to clear *Citrobacter rodentium* infection (36). Others have shown that depletion of RORγt+ T reg cells inhibited the clearance of *C. rodentium* (38). 1,25D treatments of D– mice resulted in faster clearance of *C. rodentium* infection than in D– mice, but slower clearance than D+ mice (36). Our data here suggests that the partial protection of 1,25D treated D– mice from *C. rodentium* infection could be due to the partial recovery of RORγt+ T regs [Figure 2; (36)]. The early effects of vitamin D are required to shape the microbiota and establish the colonic population of RORγt/FoxP3+ T reg cells.

T reg cells in the colon are low just after birth and increase to adult levels at 4 weeks of age (10). The temporal increase in T reg cells in the intestine with weaning and the induction of RORγt+ T reg cells in the colon with microbial colonization of GF mice demonstrates the critical role of microbes in the development of colonic T reg cells (10). The data show that intervention with 1,25D at weaning in D– mice failed to induce the microbial changes needed to increase the colonic RORγt+ T reg cells to D+ levels. Microbial exposure following birth is critical for immune development and alterations in the gut microbiota during development can have negative effects for allergy and other immune mediated diseases later in life (39). Early life colonization (before 5 weeks of age) with diverse microbes is important for the regulation of IgE; demonstrating a critical window of microbial regulation of immune cells during development (40). Like GF mice, VDR KO and D– mice have hyper IgE (41). The D– mice and GF recipients of D– microbiota that lacked RORγt+ T reg cells, *B.thetaiotaomicron* and the *Clostridium* cluster XIVa, developed more severe colitis following DSS than D+ mice or GF recipients of D+ microbiota. Others have shown that mice with reduced or absent RORγt+ T reg cells develop more severe colitis (22, 23). Vitamin D and the microbiota are both environmental factors interacting to affect immune development and the risk for immune mediated disease.

A number of individual bacterial strains have been shown to induce colonic T reg cells *in vivo*. *Clostridium ramosum* from the *Clostridium* XVIII cluster and a collection of 42 *Clostridium* species (cluster IV and XIVa) induced T reg cells in the colon of GF mice (22, 23). *B.thetaiotaomicron* has also been shown to induce colonic RORγt+ T reg cells in mice (22, 23). Induction of colonic T reg cells depends on toll-like receptor (TLR) signaling since colonization of GF mice without the TLR adapter proteins failed to induce T reg cells in the colon (42). In addition, the induction of T reg cells by *B. fragilis* required TLR2 (43). Butyrate production by microbial fermentation of fiber increased FoxP3+ T reg cells and RORγt+ T reg cells in the colon (22, 44–46). Conversely, Sefik and colleagues found no role for SCFA in the induction of RORγt+ T reg cells (23). We also did not find any significant correlation between cecal SCFA levels and RORγt+ T reg cells. Other potential mechanisms underlying the induction of RORγt+ T reg cells by the microbiota include regulation of toll like receptor signaling, induction of transforming growth factor-β, matrix metalloproteinases, and indoleamine 2,3-dioxygenase. The reduced numbers of RORγt+ T reg cells in D– and D– +1,25D treated mice corresponded to the lower levels of *Clostridium* species from cluster XIVa and *B.thetaiotaomicron*.

Bacteria do not express the VDR and therefore direct regulation of the microbiota by vitamin D seems unlikely. 1,25D treatments *in vivo* have been shown to induce peripheral T reg cells (19). The direct effects of vitamin D in the colon include regulation of immunity, barrier function, and/or the production of anti-microbial peptides (5). Vitamin D regulates innate immune responses via TLR and pattern recognition receptors (42). ILC3 cells were completely recovered in the 1,25D treated D– colon (36). ILC3 production of IL-22 depends on vitamin D in the colon and IL-22 induces antibacterial peptide production by epithelial cells (36). NOD2 a pattern recognition protein linked to the development of IBD is a vitamin D target (47). TNF-α, IL-17, and IFN-γ were all reduced in D+ as compared to D– or VDR KO mice (5, 48). Vitamin D regulates gut inflammation and the vitamin D alterations in mucosal immunity underlies the vitamin D effects on the microbial community structure.

Vitamin D status regulated the composition of the microbiota in the gut and the induction of colonic RORγt+ T reg cells. The implications of the work include that early adequate vitamin D status could determine the composition of the microbiota and early vitamin D deficiency may preclude optimal RORγt+ T reg cell expansion. Vitamin D interventions might improve vitamin D status but be ineffective for colonic RORγt+ T reg cells. Early nutrition is critical for the establishment of the community of microbes and the development of tolerance in the GI tract. Vitamin D is a micronutrient critical for optimal numbers of microbial induced RORγt+ T reg cells.

## Data Availability

The datasets generated for this study can be found in the National Center for Biotechnology Information (NCBI) the accession number PRJNA506977.

## Ethics Statement

The animal study was reviewed and approved by Institutional Animal Care and Use Committee at the Pennsylvania State University.

## Author Contributions

MC, Y-DL, SB, and AP conceptualized and designed the experimental studies. JA, Y-DL, SB, YT, and RN performed the experiments and acquired and analyzed the data. MC drafted the manuscript with the help of JA, Y-DL, YT, RN, and SB. AP critically revised the manuscript. All authors approved the publication of the manuscript.

### Conflict of Interest Statement

The authors declare that the research was conducted in the absence of any commercial or financial relationships that could be construed as a potential conflict of interest.

## References

[1] Sun M He C Cong Y Liu Z. Regulatory immune cells in regulation of intestinal inflammatory response to microbiota. Mucosal Immunol. (2015) 8:969–78. 10.1038/mi.2015.49 26080708 PMC4540654

[2] López-Muñoz P Beltrán B Sáez-González E Alba A Nos P Iborra M. Influence of vitamin D deficiency on inflammatory markers and clinical disease activity in IBD patients. Nutrients. (2019) 11:E1059. 10.3390/nu11051059 31083541 PMC6567866

[3] Cianci R Cammarota G Frisullo G Pagliari D Ianiro G Martini M. Tissue-infiltrating lymphocytes analysis reveals large modifications of the duodenal immunological niche in coeliac disease after gluten-free diet. Clin Transl Gastroenterol. (2012) 3:e28. 10.1038/ctg.2012.22 23324655 PMC3535075

[4] Zuo T Ng SC. The gut microbiota in the pathogenesis and therapeutics of inflammatory Bowel disease. Front Microbiol. (2018) 9:2247. 10.3389/fmicb.2018.02247 30319571 PMC6167487

[5] Cantorna MT McDaniel K Bora S Chen J James J. Vitamin D, immune regulation, the microbiota, and inflammatory bowel disease. Exp Biol Med. (2014) 239:1524–30. 10.1177/1535370214523890 24668555 PMC4176535

[6] Ueno A Jijon H Chan R Ford K Hirota C Kaplan GG. Increased prevalence of circulating novel IL-17 secreting Foxp3 expressing CD4+ T cells and defective suppressive function of circulating Foxp3+ regulatory cells support plasticity between Th17 and regulatory T cells in inflammatory bowel disease patients. Inflamm Bowel Dis. (2013) 19:2522–34. 10.1097/MIB.0b013e3182a85709 24097227

[7] Maul J Loddenkemper C Mundt P Berg E Giese T Stallmach A. Peripheral and intestinal regulatory CD4+ CD25(high) T cells in inflammatory bowel disease. Gastroenterology. (2005) 128:1868–78. 10.1053/j.gastro.2005.03.043 15940622

[8] Ivanov II Atarashi K Manel N Brodie EL Shima T Karaoz U. Induction of intestinal Th17 cells by segmented filamentous bacteria. Cell. (2009) 139:485–98. 10.1016/j.cell.2009.09.033 19836068 PMC2796826

[9] Gaboriau-Routhiau V Rakotobe S Lécuyer E Mulder I Lan A Bridonneau C. The key role of segmented filamentous bacteria in the coordinated maturation of gut helper T cell responses. Immunity. (2009) 31:677–89. 10.1016/j.immuni.2009.08.020 19833089

[10] Atarashi K Tanoue T Shima T Imaoka A Kuwahara T Momose Y. Induction of colonic regulatory T cells by indigenous Clostridium species. Science. (2011) 331:337–41. 10.1126/science.1198469 21205640 PMC3969237

[11] Round JL Mazmanian SK. Inducible Foxp3+ regulatory T-cell development by a commensal bacterium of the intestinal microbiota. Proc Natl Acad Sci USA. (2010) 107:12204–9. 10.1073/pnas.0909122107 20566854 PMC2901479

[12] Cantorna MT Munsick C Bemiss C Mahon BD. 1,25-Dihydroxycholecalciferol prevents and ameliorates symptoms of experimental murine inflammatory bowel disease. J Nutr. (2000) 130:2648–52. 10.1093/jn/130.11.2648 11053501

[13] Froicu M Cantorna MT. Vitamin D and the vitamin D receptor are critical for control of the innate immune response to colonic injury. BMC Immunol. (2007) 8:5. 10.1186/1471-2172-8-5 17397543 PMC1852118

[14] Froicu M Weaver V Wynn TA McDowell MA Welsh JE Cantorna MT. A crucial role for the vitamin D receptor in experimental inflammatory bowel diseases. Mol Endocrinol. (2003) 17:2386–92. 10.1210/me.2003-0281 14500760

[15] Ooi JH Li Y Rogers CJ Cantorna MT. Vitamin D regulates the gut microbiome and protects mice from dextran sodium sulfate-induced colitis. J Nutr. (2013) 143:1679–86. 10.3945/jn.113.180794 23966330 PMC3771816

[16] Bruce D Yu S Ooi JH Cantorna MT. Converging pathways lead to overproduction of IL-17 in the absence of vitamin D signaling. Int Immunol. (2011) 23:519–28. 10.1093/intimm/dxr045 21697289 PMC3139478

[17] Palmer MT Lee YK Maynard CL Oliver JR Bikle DD Jetten AM. Lineage-specific effects of 1,25-dihydroxyvitamin D(3) on the development of effector CD4 T cells. J Biol Chem. (2011) 286:997–1004. 10.1074/jbc.M110.163790 21047796 PMC3020784

[18] Kang SW Kim SH Lee N Lee WW Hwang KA Shin MS. 1,25-Dihyroxyvitamin D3 promotes FOXP3 expression via binding to vitamin D response elements in its conserved noncoding sequence region. J Immunol. (2012) 188:5276–82. 10.4049/jimmunol.1101211 22529297 PMC3358577

[19] Takiishi T Ding L Baeke F Spagnuolo I Sebastiani G Laureys J. Dietary supplementation with high doses of regular vitamin D3 safely reduces diabetes incidence in NOD mice when given early and long term. Diabetes. (2014) 63:2026–36. 10.2337/db13-1559 24550187

[20] Lochner M Peduto L Cherrier M Sawa S Langa F Varona R. *In vivo* equilibrium of proinflammatory IL-17+ and regulatory IL-10+ Foxp3+ RORgamma t+ T cells. J Exp Med. (2008) 205:1381–93. 10.1084/jem.20080034 18504307 PMC2413035

[21] Wang Y Su MA Wan YY. An essential role of the transcription factor GATA-3 for the function of regulatory T cells. Immunity. (2011) 35:337–48. 10.1016/j.immuni.2011.08.012 21924928 PMC3182399

[22] Ohnmacht C Park JH Cording S Wing JB Atarashi K Obata Y. MUCOSAL IMMUNOLOGY. The microbiota regulates type 2 immunity through RORgammat(+) T cells. Science. (2015) 349:989–93. 10.1126/science.aac4263 26160380

[23] Sefik E Geva-Zatorsky N Oh S Konnikova L Zemmour D McGuire AM. MUCOSAL IMMUNOLOGY. Individual intestinal symbionts induce a distinct population of RORgamma(+) regulatory T cells. Science. (2015) 349:993–7. 10.1126/science.aaa9420 26272906 PMC4700932

[24] Yu S Cantorna MT. Epigenetic reduction in invariant NKT cells following *in utero* vitamin D deficiency in mice. J Immunol. (2011) 186:1384–90. 10.4049/jimmunol.1002545 21191070 PMC3127168

[25] Bora SA Kennett MJ Smith PB Patterson AD Cantorna MT. The gut microbiota regulates endocrine vitamin D metabolism through fibroblast growth factor 23. Front Immunol. (2018) 9:408. 10.3389/fimmu.2018.00408 29599772 PMC5863497

[26] Chen J Waddell A Lin YD Cantorna MT. Dysbiosis caused by vitamin D receptor deficiency confers colonization resistance to *Citrobacter rodentium* through modulation of innate lymphoid cells. Mucosal Immunol. (2015) 8:618–26. 10.1038/mi.2014.94 25315967 PMC4398576

[27] Nichols RG Cai J Murray IA Koo I Smith PB Perdew GH. Structural and functional analysis of the gut microbiome for toxicologists. Curr Protoc Toxicol. (2018) 78:e54. 10.1002/cptx.54 30230220 PMC6484866

[28] Matsuki T Watanabe K Fujimoto J Kado Y Takada T Matsumoto K. Quantitative PCR with 16S rRNA-gene-targeted species-specific primers for analysis of human intestinal bifidobacteria. Appl Environ Microbiol. (2004) 70:167–73. 10.1128/AEM.70.1.167-173.2004 14711639 PMC321263

[29] Matsuki T Watanabe K Fujimoto J Takada T Tanaka R. Use of 16S rRNA gene-targeted group-specific primers for real-time PCR analysis of predominant bacteria in human feces. Appl Environ Microbiol. (2004) 70:7220–8. 10.1128/AEM.70.12.7220-7228.2004 15574920 PMC535136

[30] Tian Y Nichols RG Roy P Gui W Smith PB Zhang J Prebiotic effects of white button musroom (*Agaricus bisporus*) feeding on succinate and intestinal gluconeogenesis in C57BL/6 mice. J Funct Foods. (2018) 45:223–32. 10.1016/j.jff.2018.04.008

[31] Tian Y Nichols RG Cai J Patterson AD Cantorna MT. Vitamin A deficiency in mice alters host and gut microbial metabolism leading to altered energy homeostasis. J Nutr Biochem. (2018) 54:28–34. 10.1016/j.jnutbio.2017.10.011 29227833 PMC5866754

[32] Kozich JJ Westcott SL Baxter NT Highlander SK Schloss PD. Development of a dual-index sequencing strategy and curation pipeline for analyzing amplicon sequence data on the MiSeq Illumina sequencing platform. Appl Environ Microbiol. (2013) 79:5112–20. 10.1128/AEM.01043-13 23793624 PMC3753973

[33] Das S Batra SK Rachagain S Mouse model of dextran sodium sulfate (DSS)-induced colitis. Bio-protocol. (2017) 7:e2515 10.21769/BioProtoc.2515 PMC8413514 34541176

[34] Ooi JH Waddell A Lin YD Albert I Rust LT Holden V. Dominant effects of the diet on the microbiome and the local and systemic immune response in mice. PLoS ONE. (2014) 9:e86366. 10.1371/journal.pone.0086366 24489720 PMC3906035

[35] Chen J Bruce D Cantorna MT. Vitamin D receptor expression controls proliferation of naive CD8+ T cells and development of CD8 mediated gastrointestinal inflammation. BMC Immunol. (2014) 15:6. 10.1186/1471-2172-15-6 24502291 PMC3923390

[36] Lin Y Arora J Diehl K Bora S Cantorna MT Vitamin D is required for ILC3 derived IL22 and protection from *Citrobacter rodentium* infection. Front Immunol. (2019) 10:1 10.3389/fimmu.2019.00001 30723466 PMC6349822

[37] Atarashi K Tanoue T Oshima K Suda W Nagano Y Nishikawa H. Treg induction by a rationally selected mixture of *Clostridia* strains from the human microbiota. Nature. (2013) 500:232–6. 10.1038/nature12331 23842501

[38] Wang Z Friedrich C Hagemann SC Korte WH Goharani N Cording S. Regulatory T cells promote a protective Th17-associated immune response to intestinal bacterial infection with *C. rodentium*. Mucosal Immunol. (2014) 7:1290–301. 10.1038/mi.2014.17 24646939

[39] Tanaka M Nakayama J. Development of the gut microbiota in infancy and its impact on health in later life. Allergol Int. (2017) 66:515–22. 10.1016/j.alit.2017.07.010 28826938

[40] Cahenzli J Koller Y Wyss M Geuking MB McCoy KD. Intestinal microbial diversity during early-life colonization shapes long-term IgE levels. Cell Host Microbe. (2013) 14:559–70. 10.1016/j.chom.2013.10.004 24237701 PMC4049278

[41] James J Weaver V Cantorna MT. Control of circulating IgE by the vitamin D receptor *in vivo* involves B cell intrinsic and extrinsic mechanisms. J Immunol. (2017) 198:1164–71. 10.4049/jimmunol.1601213 28003380 PMC5263170

[42] Geuking MB Cahenzli J Lawson MA Ng DC Slack E Hapfelmeier S. Intestinal bacterial colonization induces mutualistic regulatory T cell responses. Immunity. (2011) 34:794–806. 10.1016/j.immuni.2011.03.021 21596591

[43] Round JL Lee SM Li J Tran G Jabri B Chatila TA. The Toll-like receptor 2 pathway establishes colonization by a commensal of the human microbiota. Science. (2011) 332:974–7. 10.1126/science.1206095 21512004 PMC3164325

[44] Smith PM Howitt MR Panikov N Michaud M Gallini CA Bohlooly-Y M. The microbial metabolites, short-chain fatty acids, regulate colonic Treg cell homeostasis. Science. (2013) 341:569–73. 10.1126/science.1241165 23828891 PMC3807819

[45] Furusawa Y Obata Y Fukuda S Endo TA Nakato G Takahashi D. Commensal microbe-derived butyrate induces the differentiation of colonic regulatory T cells. Nature. (2013) 504:446–50. 10.1038/nature12721 24226770

[46] Arpaia N Campbell C Fan X Dikiy S van der Veeken J deRoos P. Metabolites produced by commensal bacteria promote peripheral regulatory T-cell generation. Nature. (2013) 504:451–5. 10.1038/nature12726 24226773 PMC3869884

[47] Wang TT Dabbas B Laperriere D Bitton AJ Soualhine H Tavera-Mendoza LE. Direct and indirect induction by 1,25-dihydroxyvitamin D3 of the NOD2/CARD15-defensin beta2 innate immune pathway defective in Crohn disease. J Biol Chem. (2010) 285:2227–31. 10.1074/jbc.C109.071225 19948723 PMC2807280

[48] Froicu M Zhu Y Cantorna MT. Vitamin D receptor is required to control gastrointestinal immunity in IL-10 knockout mice. Immunology. (2006) 117:310–8. 10.1111/j.1365-2567.2005.02290.x 16476050 PMC1782241

