# Supplementary material for: Vitamin D Regulates the Microbiota to Control the Numbers of RORγt/FoxP3+ Regulatory T Cells in the Colon
Source: Front Immunol. 2019 Jul 30;10:1772. doi: 10.3389/fimmu.2019.01772 (PMC6682671; doi:10.3389/fimmu.2019.01772)
Supplement: Supplementary file 1 [file Data_Sheet_1.pdf]

Vitamin D regulates the microbiota to induce RORγt/FoxP3+ regulatory T cells

Margherita T. Cantorna[1,2],Yang-Ding Lin[1,2], Juhi Arora[1,2], Stephanie Bora,[1,2,4] Yuan Tian[1,2,3], Robert G. Nichols[1], and Andrew D. Patterson[1]

[1]Department of Veterinary and Biomedical Science, The Pennsylvania State University, University Park, PA 16802, USA

[2]Center for Molecular Immunology and Infectious Disease, The Pennsylvania State University, University Park, PA 16802, USA

Correspondence to: Dr. Margherita T. Cantorna, Department of Veterinary and Biomedical Sciences, The Center for Molecular Immunology and Infectious Disease, 115 Henning Bldg., University Park, PA, 16802, USA.

Supplementary Methods

^{1}H NMR spectroscopy and data processing. The cecal content were prepared for NMR as previously described (34). ^{1}H NMR spectra were recorded at 298 K on a Bruker Avance III 600 MHz spectrometer equipped with an inverse cryogenic probe (Bruker Biospin, Germany). NMR spectra from cecal samples were acquired individually employing the first increment of NOESY pulse sequence (NOESYPR1D). For accurate quantification of SCFA, a delay was added in the addition to recycle delay (RD) in the sequence of cecal content samples (2). ^{1}H NMR spectra were corrected for phase and baseline using TOPSPIN (V3.5, Bruker Biospin). Each bucketed region (0.004 ppm) was then normalized to the total sum of the spectral integrals prior to statistical data analysis. Orthogonal projection to latent structure-discriminant analysis (OPLS-DA) were carried out using the SIMCA-P+ software (Version 13.0, Umetrics, Sweden). The quality of the model was described by the parameters R^2X and Q^2. The validity of the OPLS-DA model was further assessed with CV-ANOVA tests for significance and $P< 0.05$ (35). Back-transformed loadings

from the OPLS-DA models were performed with color-coded correlation coefficient for variables, using an in-house developed script for MATLAB (The Mathworks Inc., Natwick, MA). The NMR data is available at the metobolomics workbench at http://www.metabolomicsworkbench.org with the data track id 1775.

STable 1. 16S rRNA gene-targeted group-specific primers

Bacterial group	Sequence (5'-3')	Reference
Universal	ACT CCT ACG GGA GGC AGC AG	(1)
	ATT ACC GCG GCT GCT GG	
Firmicutes	GCA GTA GGG AAT CTT CCG	(1)
	ATT ACC GCG GCT GCT GG	
Bacteroidetes	GTA CTG AGA CAC GGA CCA	(1)
	ATT ACC GCG GCT GCT GG	
Bacteroides fragilis	ATA GCC TTT CGA AAG RAA GAT	(2)
	CCA GTA TCA ACT GCA ATT TTA	
Bacteroides thetaiotaomicron	GCA AAC TGG AGA TGG CGA	(3)
	AAG GTT TGG TGA GCC GTTA	
Clostridium XIVa	AAA TGA CGG TAC CTG ACT AA	(2)
	CTT TGA GTT TCA TTC TTG CGA A	
Clostridium IV	GCA CAA GCA GTG GAG T	(2)
	CTT CCT CCG TTT TGT CAA	
Clostridium XVIII	GTG ACC GTA TTA AAA GTG CCT	(4)
	TAC CGT CAC TCG GCT AC	

1. Fierer, N., J. A. Jackson, R. Vilgalys, and R. B. Jackson. 2005. Assessment of soil microbial community structure by use of taxon-specific quantitative PCR assays. *Appl Environ Microbiol* 71: 4117-4120.
2. Matsuki, T., K. Watanabe, J. Fujimoto, T. Takada, and R. Tanaka. 2004. Use of 16S rRNA gene-targeted group-specific primers for real-time PCR analysis of predominant bacteria in human feces. *Appl Environ Microbiol* 70: 7220-7228.
3. Narushima, S., Y. Sugiura, K. Oshima, K. Atarashi, M. Hattori, M. Suematsu, and K. Honda. 2014. Characterization of the 17 strains of regulatory T cell-inducing human-derived Clostridia. *Gut microbes* 5: 333-339.
4. Matsuki, T., K. Watanabe, J. Fujimoto, Y. Miyamoto, T. Takada, K. Matsumoto, H. Oyaizu, and R. Tanaka. 2002. Development of 16S rRNA-gene-targeted group-specific primers for the detection and identification of predominant bacteria in human feces. *Appl Environ Microbiol* 68: 5445-5451.

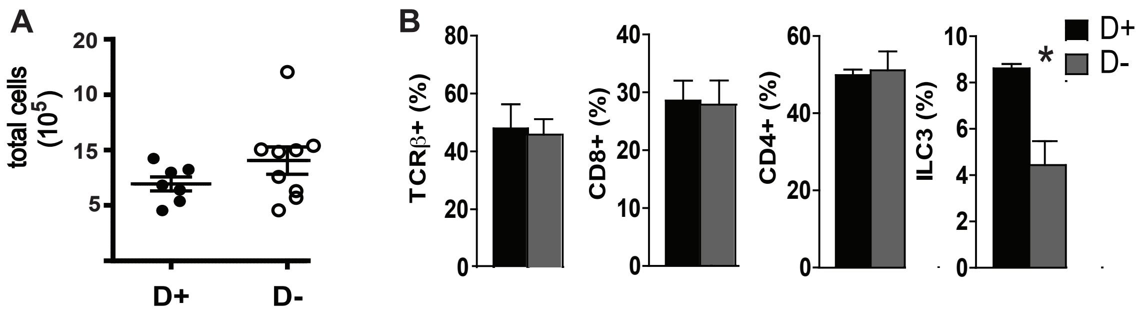

Supplemental figure 1. The lymphocytes in the colon lamina propria of D+ and D- mice were isolated. A) The total cell numbers isolated from the D+ and D- colon lamina propria and B) TCRβ, CD8, CD4 and ILC frequencies are reported. Values are the mean +/- SEM from n=4-8 mice per group from two independent experiments.*P<0.05.

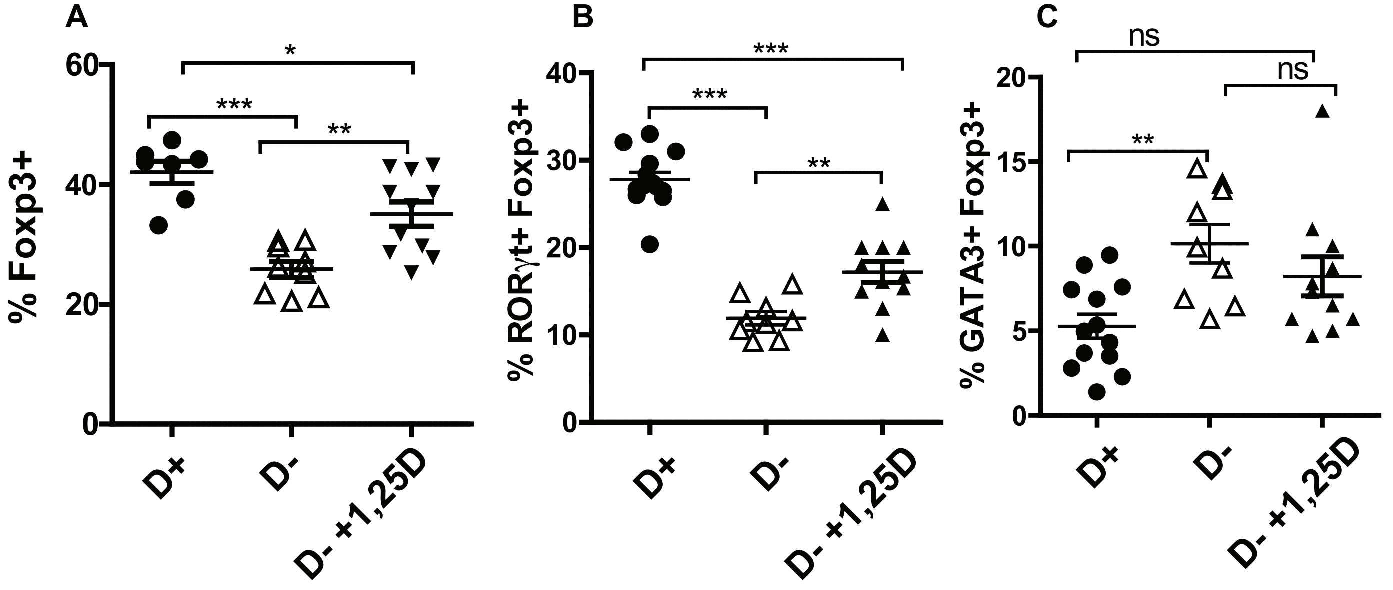

Supplementary figure 2. 1,25D treatments of D- mice was started at 5 wks of age and continued until the mice were sacrificed at 8 wks of age. **A)** Frequencies of FoxP3+ T reg, **B)** RORγt/FoxP3+ T reg, and **C)** GATA3/FoxP3+ T reg. Values are mean ± SEM of two combined experiments and n= 7-13 mice/ group. One-way ANOVA and Bonferroni post tests, *P<0.05, **P<0.01, ***P<0.001.

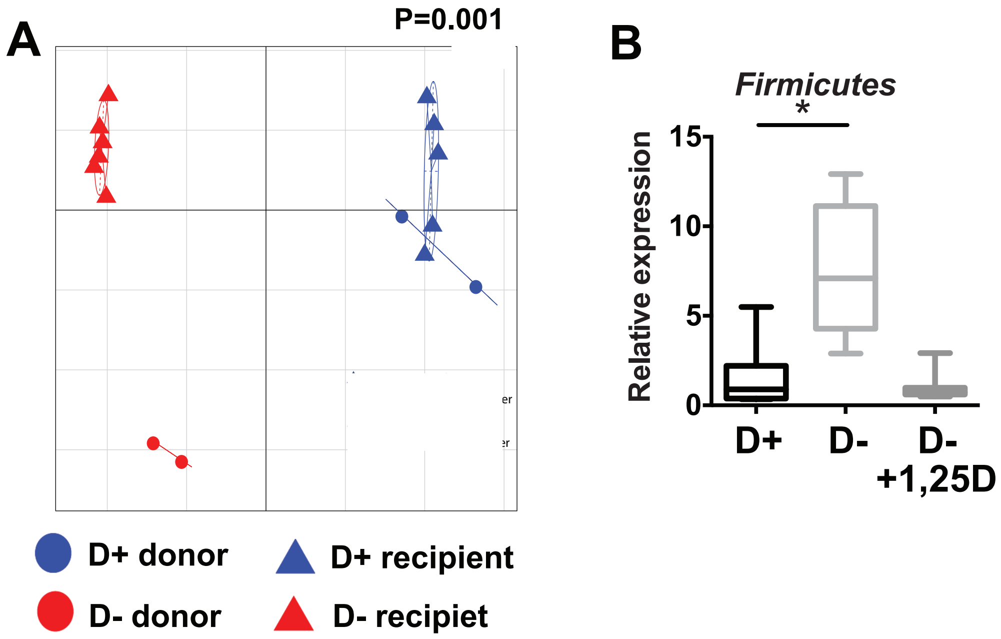

Supplemental figure 3. A) Microbial communities in D+ and D- donor and recipients. Generalized Unifrac analysis of the total population of microbes: significance was determined using Permutation MANOVA with the R package adonis.2. B) qPCR analysis of the proportion of Firmicutes in the cecum of D+, D- and D- +1,25D treated mice. Values are the mean ± SEM two combined experiments and n= 6-14 mice/group. Significance was determined using one-way ANOVA with Kruskal-Wallis post-hoc tests, *P<0.05.

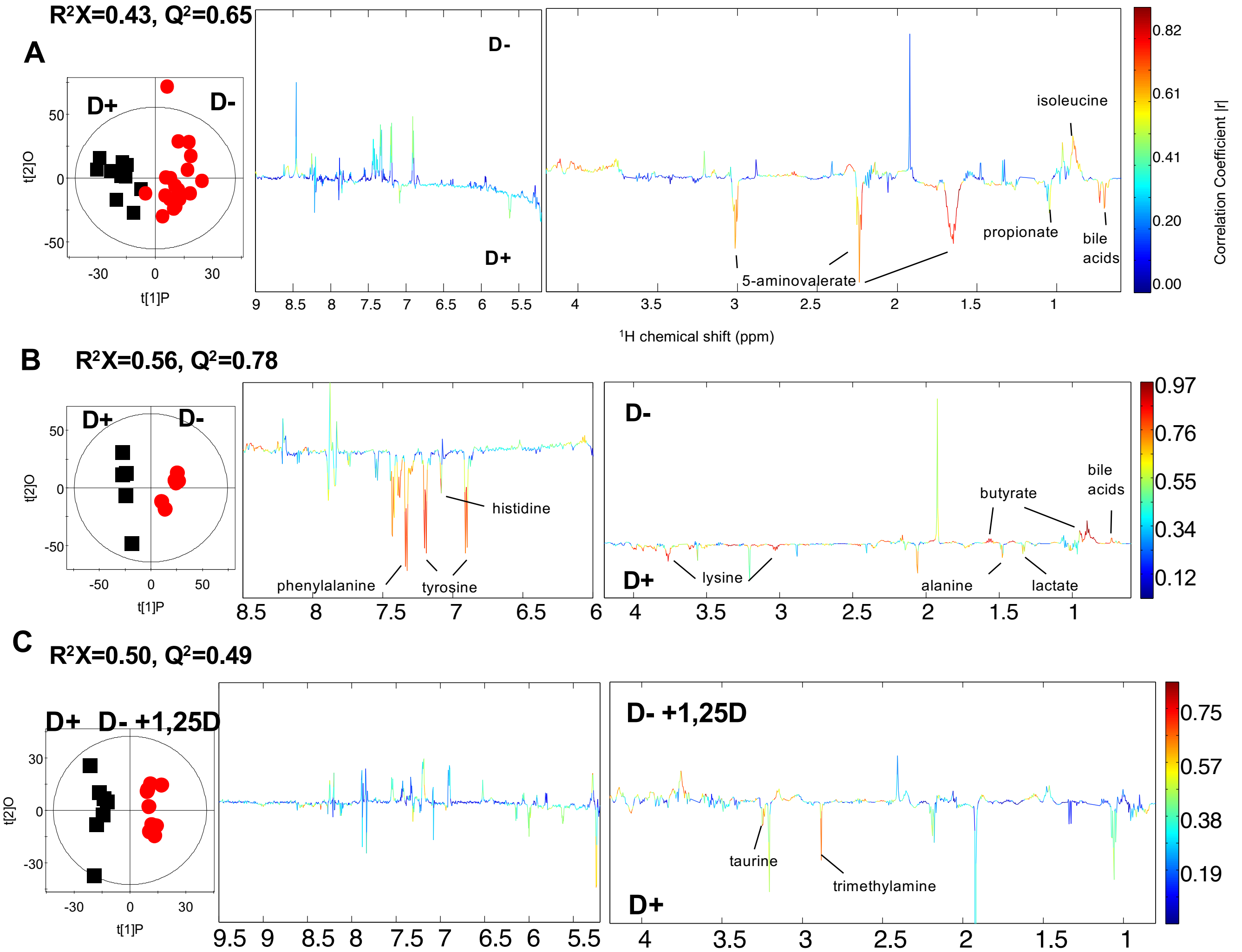

Supplementary figure 4. Cecal metabolites. ^{1}H NMR spectra of cecal contents. OPLS-DA scores plot (left) and coefficient plot (right) derived from ^{1}H NMR spectra of cecal contents. **A)** Donor D+ (■) and donor D- (●) mice, **B)** recipient D+ (■) and recipient D- (●) mice, and C) D+ (■) and D- +1,25D (●). Each model was evaluated with CV-ANOVA with (A) P= 5 E-5 and n = 10-18 mice/group, (B) P= 4 E-4 and n = 5-6 mice/group and (C) P= 0.02 and n = 8-10 mice/group.

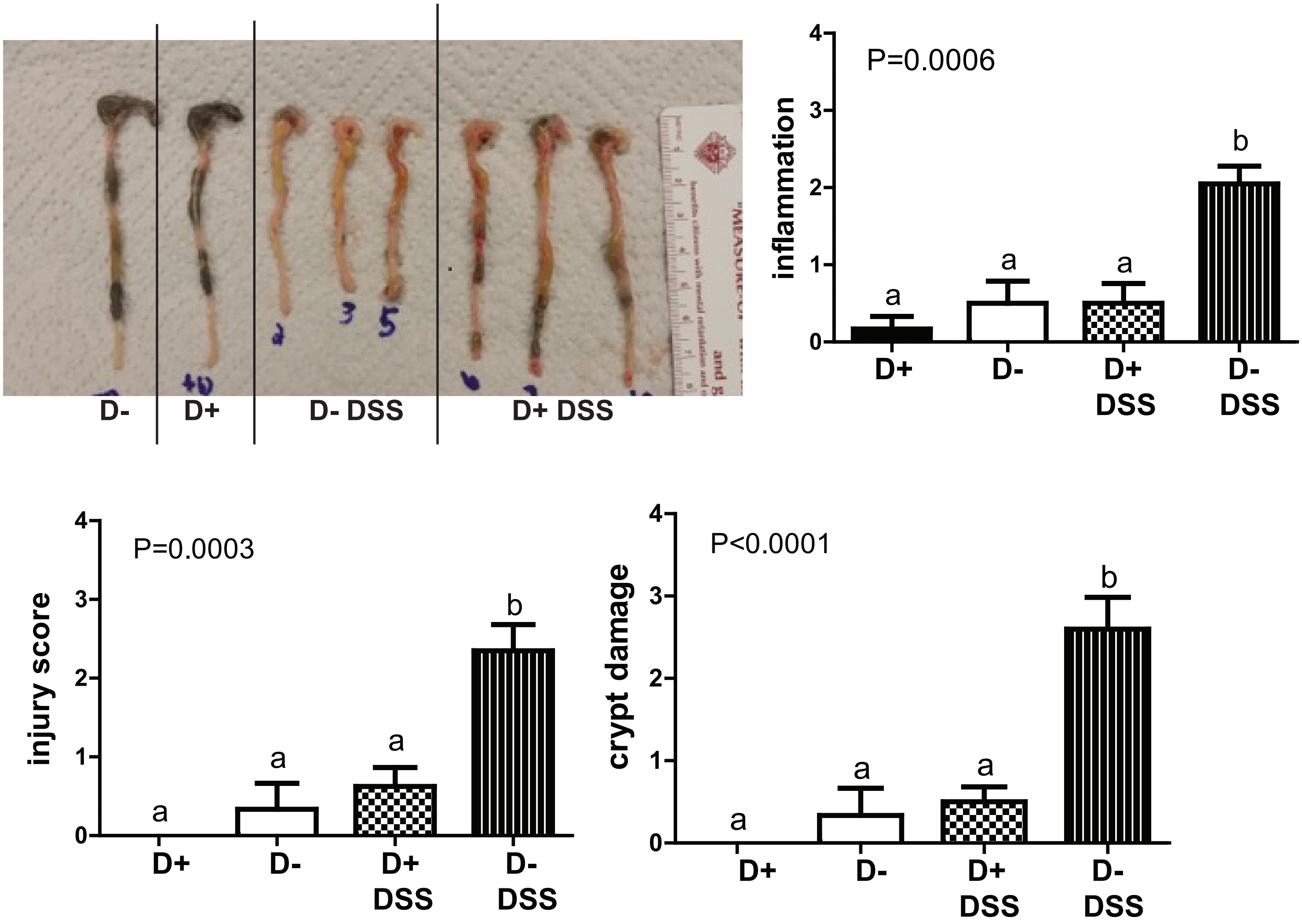

Supplemental figure 5: Pictures of the colons of 1 untreated D- and D+ mouse and 3 DSS treated D- and D+ mice at day 10 post-DSS. Histopathology scores for inflammation, injury, and crypt damage in untreated D+, and D mice or day 10 post-DSS, D+ DSS, and D- DSS mice. Values are the mean + SEM of n=3-6 mice per group. One-way ANOVA with Tukey's multiple comparison was used to assess signficance. Values with different letters were significantly different with the indicated P values.
